# Development and validation of a nomogram to predict cardiac death after radiotherapy for esophageal cancer

**DOI:** 10.1002/cai2.89

**Published:** 2023-09-01

**Authors:** Xinfang Lv, Xue Wu, Kai Liu, Xinke Zhao, Chenliang Pan, Jing Zhao, Juan Chang, Huan Guo, Xiang Gao, Xiaodong Zhi, Chunzhen Ren, Qilin Chen, Hugang Jiang, Chunling Wang, Ying‐Dong Li

**Affiliations:** ^1^ Department of Geriatrics Affiliated Hospital of Gansu University of Traditional Chinese Medicine Lanzhou Gansu China; ^2^ School of Integrative Medicine, Gansu University of Chinese Medicine Lanzhou Gansu China; ^3^ Department of Cardiology The Second Hospital of Lanzhou University Lanzhou Gansu China; ^4^ Cardiovascular Disease Center, The First Hospital of Lanzhou University Lanzhou Gansu China; ^5^ Department of Traditional Medicine Gansu Provincial Hospital Lanzhou Gansu China; ^6^ Center for Translational Medicine, Gansu Provincial Academic Institute for Medical Research Lanzhou Gansu China

**Keywords:** cardiac death, cardio‐oncology, esophageal cancer, nomogram, radiotherapy

## Abstract

**Background:**

Patients frequently die from cardiac causes after radiotherapy for esophageal cancer. Early detection of cardiac death risk in these patients is crucial to improve clinical decision‐making and prognosis. Thus, we modeled the risk of cardiac death after irradiation for esophageal cancer.

**Methods:**

A retrospective analysis of 37,599 esophageal cancer cases treated with radiotherapy in the SEER database between 2000 and 2018 was performed. The selected cases were randomly assigned to the model development group (*n* = 26,320) and model validation group (*n* = 11,279) at a ratio of 7:3. We identified the risk factors most commonly associated with cardiac death by least absolute shrinkage and selection operator regression analysis (LASSO). The endpoints for model development and validation were 5‐ and 10‐year survival rates. The net clinical benefit of the models was evaluated by decision curve analysis (DCA) and concordance index (C‐index). The performance of the models was further assessed by creating a receiver operating characteristic curve (ROC) and calculating the area under the curve (AUC). Kaplan‐Meier (K‐M) survival analysis was performed on the probability of death. Patients were classified according to death probability thresholds. Five‐ and ten‐year survival rates for the two groups were shown using K‐M curves.

**Results:**

The major risk factors for cardiac death were age, surgery, year of diagnosis, sequence of surgery and radiotherapy, chemotherapy and a number of tumors, which were used to create the nomogram. The C‐indexes of the nomograms were 0.708 and 0.679 for the development and validation groups, respectively. DCA showed the good net clinical benefit of nomograms in predicting 5‐ and 10‐year risk of cardiac death. The model exhibited moderate predictive power for 5‐ and 10‐year cardiac mortality (AUC: 0.833 and 0.854, respectively), and for the development and validation cohorts (AUC: 0.76 and 0.813, respectively).

**Conclusions:**

Our nomogram may assist clinicians in making clinical decisions about patients undergoing radiotherapy for esophageal cancer based on early detection of cardiac death risk.

## Graphical abstract

Radiotherapy for esophageal cancer (EC) increased cardiovascular toxicity and mortality in patient d. We developed and validated a nomogram that accurately predicted cardiac mortality variables in EC patients after radiotherapy. Risk factors such as age, year of diagnosis, surgery, sequence of surgery and radiotherapy, chemotherapy, and a number of tumors were included in the nomogram.

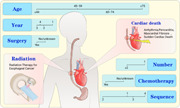

**Abbreviations**
5‐FU: 5‐fluorouracil
ACS: acute coronary syndrome
AD: autonomic dysfunction
ANOVA: analysis of variance
AUC: area under the curve
C‐index: concordance index
DCA: decision curve analysis
DDP: cisplatin
Dox: doxorubicin
EC: esophageal cancer
EGFR: epidermal growth factor receptor
ESC: European Society of Cardiology
HER‐2: human epidermal growth factor receptor 2
HF: heart failure
IRAEs: immune‐related adverse events
ROC: receiver operating characteristic
VEGFR: vascular endothelial growth factor receptor

## 1 BACKGROUND

In terms of mortality, esophageal cancer (EC) ranks sixth among cancers worldwide and seventh in incidence [1]. Chemotherapy, immunotherapy, and radiotherapy are the main nonsurgical treatments for EC, but all of them can cause cardiotoxicity to varying degrees. Cardiovascular disease is the second leading cause of death in patients with EC [2, 3]. The most commonly used chemotherapeutic drugs for EC include cisplatin (DDP), 5‐fluorouracil (5‐FU), and doxorubicin (Dox) [4]. Cisplatin produces cardiotoxicity through DNA damage, ROS‐mediated oxidative stress, inflammation, and MAPK signaling pathway activation [5]; 5‐FU leads to activation of protein kinase C, increased nitric oxide, imbalance of oxygen supply and demand, and cardiac arterial thrombosis, and the direct cardiotoxic effects lead to coronary toxicity, arrhythmia, blood pressure changes and even cardiogenic shock [6, 7, 8]; Dox causes cumulative and dose‐dependent cardiotoxicity through oxidative stress and apoptosis [9]. Immunotherapy is a major advance in cancer theatment, although the reported incidence of immune‐related cardiovascular events is 1.14%–5% (including myocarditis 0.3%–1.4%, arrhythmia 3.6%–4.8%, pericardial disease 1.74%, vasculitis 0.27%, acute coronary syndrome (ACS) 0.95%–7.0% and heart failure (HF) 1.6%), the mortality rate can be as high as 50%, and the possible etiology of cardiovascular immune‐related adverse events (IRAE) includes local T cell activation, cross‐reactivity of antitumor T cells with myocardial antigens, or systemic immune activation [10]. In addition, targeted therapy targeting epidermal growth factor receptor (EGFR), human epidermal growth factor receptor 2 (HER‐2), vascular endothelial growth factor receptor (VEGFR), and other key signaling pathways and epigenetic targeted therapy is also evolving, but the attendant cardiotoxicity is also a concern [11].

Radiation‐induced cardiac complications mainly include pericardial disease, cardiomyopathy, coronary atherosclerosis, valvular heart disease, and arrhythmia. The overall incidence of symptomatic cardiotoxicity during radiation therapy for EC was as high as 10.8%, and the most common complications were pericardial effusion, ischemic heart disease, and HF [12, 13, 14]. The mechanism of myocardial injury induced by radiotherapy is related to microvascular alterations and inflammation leading to longer‐term fibrotic changes, endothelial cell changes leading to a decrease in the ratio of capillaries to cardiomyocytes, epicardial vessel damage leading to a prothrombotic state, and activation of inflammatory proteins leading to persistent inflammation and autonomic dysfunction (AD) [15]. Several recent studies [16, 17, 18, 19, 20, 21, 22] have shown that the cardiotoxicity and severity of EC increase with radiation dose, age, and postradiation time. There is limited research on radiotherapy and cardiovascular mortality in EC, and a study by Frandsen et al. [23] showed that EC patients treated with radiotherapy were at higher risk of cardiac‐related death, with absolute risks of cardiac death of 2.8%, 5.3%, and 9.4% at 5, 10, and 20 years, respectively. A study by Gharzai et al. [24] also showed that EC patients who received radiotherapy had a higher risk of cardiac death than those who did not.

**Figure 1:**
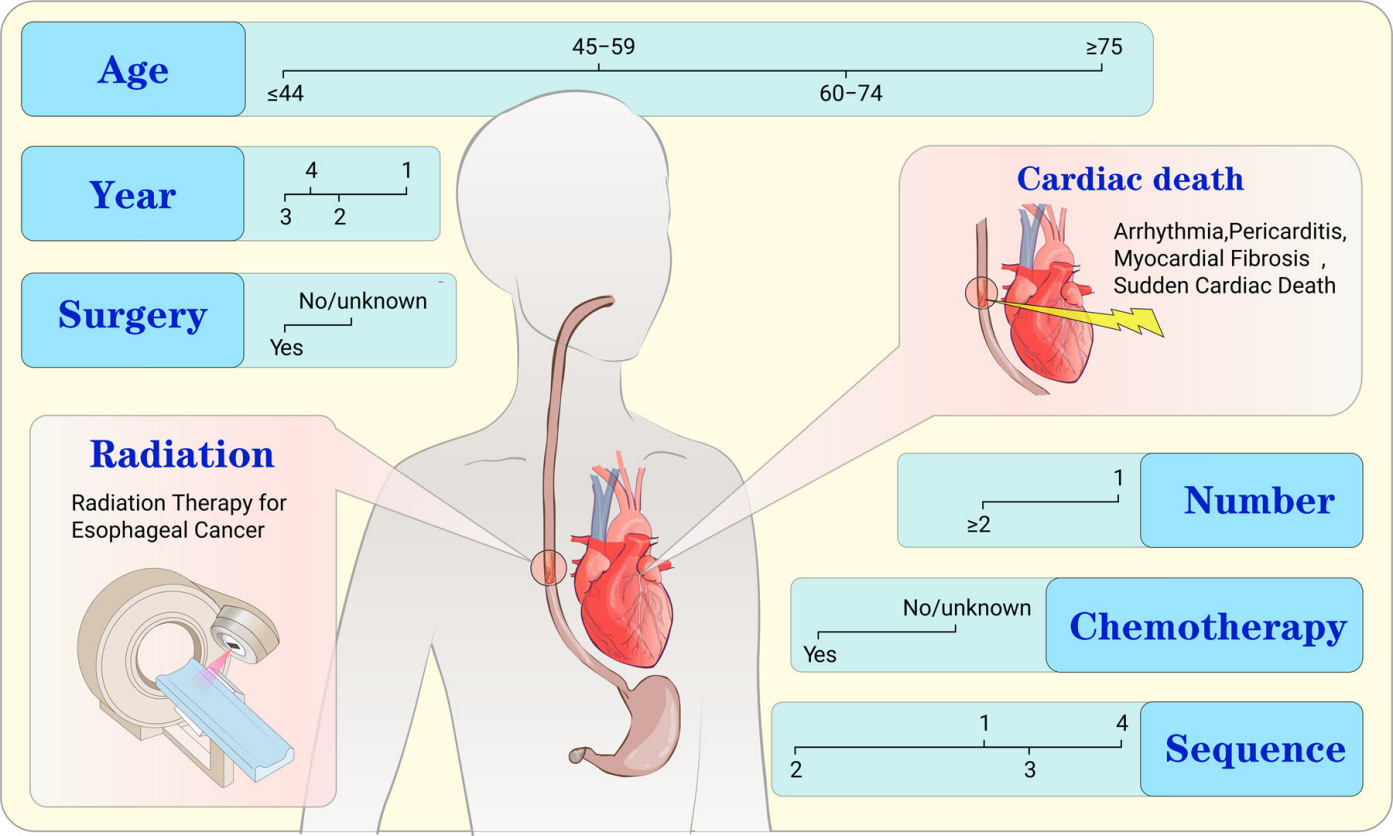
Graphical summary. Esophageal cancer (EC) radiotherapy increased cardiovascular toxicity and mortality in patients. A nomogram was developed and validated to accurately predict cardiac death in EC patients after radiotherapy. Risk factors such as age, year of diagnosis, surgery, sequence of surgery and radiotherapy, chemotherapy, and number of tumors are included in the nomogram.

The European Society of Cardiology (ESC) recommends a baseline cardiovascular risk assessment for all patients receiving thoracic radiotherapy and an annual cardiovascular risk assessment for all cancer survivors who have previously received thoracic radiotherapy or cardiotoxic drugs [25]. Cardiovascular toxicity associated with cancer therapy could be detected in a timely manner. In addition, there is a need for a clinical predictive model capable of early identification of the risk of cardiac death in EC patients receiving radiotherapy. Nomograms are of high clinical value by transforming complex regression equations into visual charts, making the results easier to read and easier for doctors to assess the patients' conditions [26, 27]. Therefore, this study aims to develop a nomogram model that can predict the 5‐ and 10‐year risk of cardiac death in patients undergoing EC radiotherapy to assist early clinical identification of high‐risk groups (Figure 1).

## 2 METHODS

### 2.1 Data sources

Information on patients with EC who received radiotherapy between 2000 and 2018 was obtained for the current study using SEER*Stat Software version 8.4.0. The SEER database records cancer incidence, death, and prevalence for approximately one‐third of US citizens. Clinical information on patient demographics, original tumor site, tumor morphological characteristics, diagnostic grade, initial treatment, and follow‐up survival status were regularly collected and added to the database.

### 2.2 Patient selection

This was a retrospective study, and cases meeting the following criteria were included in the analysis:

(1) Site recode: esophagus.
(2) Primary sites include cervical esophagus (C15.0), thoracic esophagus (C15.1), abdominal esophagus (C15.2), upper third, middle, and lower third of the esophagus (C15.3), overlapping esophageal lesions (C15.8) esophagus (C15.9) and not otherwise specified (NOS).
(3) Received beam radiation, radioactive implant radiation (including brachytherapy), radioisotope radiation, beam plus implant or isotope radiation, and NOS radiation.
(4) Cases with zero follow‐up time and cases with an undetermined total number of malignancies or tumors in situ were excluded.

Age, sex, race, marital status at diagnosis, year of diagnosis, primary site, ICD‐O‐3 histology code (hist)/behavior (behav), a total number of malignancies, tumor size, grade and TNM stage, surgical procedure, sequence of surgery and radiotherapy, type of radiation therapy, chemotherapy, survival status, and cause of death were extracted for each patient.

### 2.3 Statistical analysis

The finally included cases were randomly divided into a model development group and a model validation group at a ratio of 7:3. Based on least absolute shrinkage and selection operator (LASSO) regression analysis, we identified the leading causes of cardiac death after EC radiation treatment. Nomograms were constructed from the LASSO regression of the team responsible for the development, using 5‐ and 10‐year survival rates as endpoints. Comparisons between risk factors were performed using analysis of variance (ANOVA).

The performance of models was evaluated using the concordance index (C‐index) as a predictor. C‐index measures the predictive effectiveness of the model. Calibration curves were calculated using the R packages “rms,” “foreign,” and “survival”. The clinical benefits of the nomograms of 5‐ and 10‐years were assessed using decision curve analysis (DCA). The likelihood of cardiac death in patients receiving radiation for EC was calculated using Cox proportional hazards regression analysis based on model components. The values of area under the curve (AUC) were based on the receiver operating characteristic curve (ROC) curves. The Kaplan‐Meier (K‐M) survival curves were plotted for the two groups of patients, and the patients were divided into two groups according to the probability of death. Statistical analyses were performed using R 4.0.5 (https://cran.r-project.org).

## 3 RESULTS

### 3.1 Patient characteristics overview

**Figure 2:**
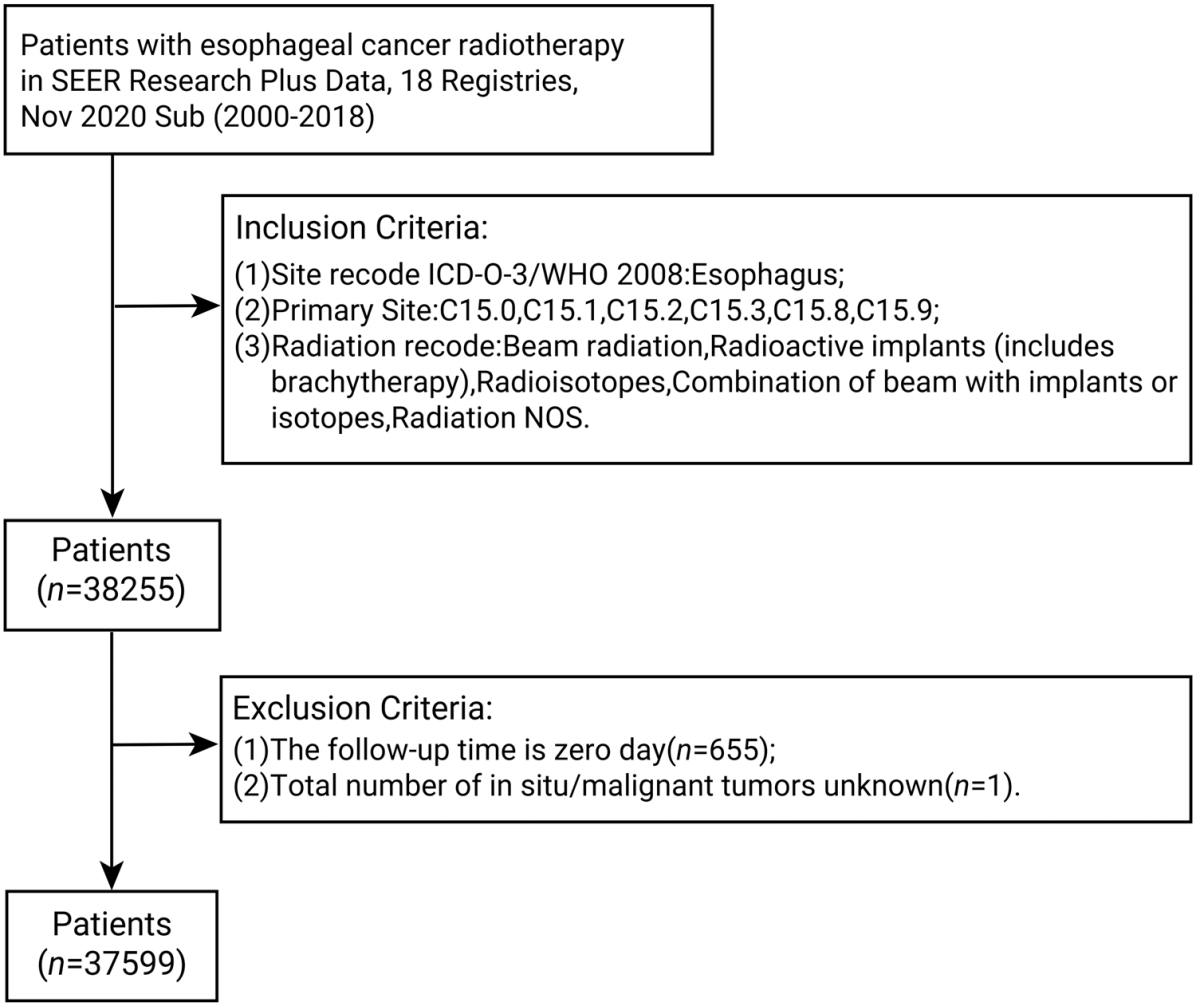
Flowchart of patient selection.

A flow chart of the inclusion process for this study is shown in Figure 2. This study included 37,599 individuals, of whom 26,320 (70%) belonged to the model development group, and 11,279 (30%) belonged to the model validation group. As shown in Table 1, there was no statistical difference between the two groups (*p* > 0.05). The most common sites, pathological types and tumor grades were thoracic esophagus (C15.1), adenocarcinoma, and grades II and III, respectively. More than 70% of the patients did not receive surgery, and most of them were treated with combination radiotherapy and chemotherapy. The most common number of tumors was one. Mean follow‐up in the two groups was 193 and 199 months, respectively.

**Table 1:** The baseline characteristics of patients in the development and validation groups.

Characteristics	Level	All patients (*n* = 37599)	Development group (*n* = 26320)	Validation group (*n* = 11279)	*p*‐value
Age, *n* (%)	≤44	973 (2.59)	704 (2.67)	269 (2.38)	0.445
	45–59	9316 (24.78)	6509 (24.73)	2807 (24.89)	
	60–74	17452 (46.41)	12206 (46.38)	5246 (46.51)	
	≥75	9858 (26.22)	6901 (26.22)	2957 (26.22)	
Sex, *n* (%)	Male	29447 (78.32)	20603 (78.28)	8844 (78.41)	0.7859
	Female	8152 (21.68)	5717 (21.72)	2435 (21.59)	
Race, *n* (%)	White	31078 (82.66)	21749 (82.63)	9329 (82.71)	0.7148
	Black	4398 (11.70)	3069 (11.66)	1329 (11.78)	
	Others	2123 (5.64)	1502 (5.71)	621 (5.51)	
Marital status, *n* (%)	Married	21441 (57.03)	14958 (56.83)	6483 (57.48)	0.1554
	Single	5595 (14.88)	3977 (15.11)	1618 (14.35)	
	Others	10563 (28.09)	7385 (28.06)	3178 (28.17)	
Year, *n* (%)	2000–2005	10753 (28.60)	7544 (28.66)	3209 (28.45)	0.606
	2006–2010	9560 (25.43)	6701 (25.46)	2859 (25.35)	
	2011–2015	10671 (28.38)	7490 (28.46)	3181 (28.20)	
	2016–2018	6615 (17.59)	4585 (17.42)	2030 (18.00)	
Site, *n* (%)	Cervical esophagus	1021 (2.72)	735 (2.79)	286 (2.54)	0.7069
	Thoracic esophagus	32294 (85.89)	22586 (85.81)	9708 (86.07)	
	Abdominal esophagus	260 (0.69)	179 (0.68)	81 (0.72)	
	Overlapping esophagus lesion	1618 (4.30)	1134 (4.31)	484 (4.29)	
	Others	2406 (6.40)	1686 (6.41)	720 (6.38)	
Histological type, *n* (%)	Squamous cell carcinoma	14421 (38.35)	10107 (38.40)	4314 (38.25)	0.5617
	Adenocarcinoma	20209 (53.75)	14112 (53.62)	6097 (54.05)	
	Others	2969 (7.90)	2101 (7.98)	868 (7.70)	
Grade, *n* (%)	I	1460 (3.88)	1039 (3.95)	421 (3.73)	0.2436
	II	12318 (32.76)	8530 (32.41)	3788 (33.58)	
	III	14784 (39.32)	10390 (39.48)	4394 (38.96)	
	IV	515 (1.37)	362 (1.38)	153 (1.36)	
	Unknown	8522 (22.67)	5999 (22.78)	2523 (22.37)	
T, *n* (%)	T1	2239 (5.96)	1578 (6.00)	661 (5.86)	0.7375
	T2	1591 (4.23)	1123 (4.27)	468 (4.15)	
	T3	5509 (14.65)	3888 (14.77)	1621 (14.37)	
	T4	1342 (3.57)	930 (3.53)	412 (3.65)	
	Unknown	26918 (71.59)	18801 (71.43)	8117 (71.97)	
N, *n* (%)	N0	4446 (11.83)	3128 (11.88)	1318 (11.69)	0.6222
	N1	5615 (14.93)	3968 (15.08)	1647 (14.60)	
	N2	1447 (3.85)	1000 (3.80)	447 (3.96)	
	N3	433 (1.15)	297 (1.13)	136 (1.21)	
	Unknown	25658 (68.24)	17927 (68.11)	7731 (68.54)	
M, *n* (%)	M0	9769 (25.98)	6822 (25.92)	2947 (26.13)	0.3888
	M1	2840 (7.56)	2020 (7.67)	820 (7.27)	
	Unknown	24990 (66.46)	17478 (66.41)	7512 (66.60)	
Surgery, *n* (%)	Yes	9629 (25.61)	6693 (25.43)	2936 (26.03)	0.2258
	No/unknown	27970 (74.39)	19627 (74.57)	8343 (73.97)	
Sequence, *n* (%)	Radiation prior to surgery	7606 (20.23)	5264 (20.00)	2342 (20.76)	0.3228
	Intraoperative radiation	372 (0.99)	257 (0.98)	115 (1.02)	
	Radiation after surgery	3090 (8.22)	2185 (8.30)	905 (8.03)	
	Unknown	26531 (70.56)	18614 (70.72)	7917 (70.19)	
Radiation, *n* (%)	Beam radiation	36669 (97.53)	25687 (97.59)	10982 (97.36)	0.4281
	Combination of beam radiation with implants or isotopes	173 (0.46)	121 (0.46)	52 (0.46)	
	Radioisotopes and radioactive implants	104 (0.28)	74 (0.28)	30 (0.27)	
	Others	653 (1.73)	438 (1.67)	215 (1.91)	
Chemotherapy, *n* (%)	Yes	31554 (83.92)	22111 (84.01)	9443 (83.72)	0.4981
	No/unknown	6045 (16.08)	4209 (15.99)	1836 (16.28)	
Number, *n* (%)	1	28203 (75.01)	19715 (74.91)	8488 (75.25)	0.4808
	≥2	9396 (24.99)	6605 (25.09)	2791 (24.75)	

### 3.2 Risk factors identification

**Figure 3:**
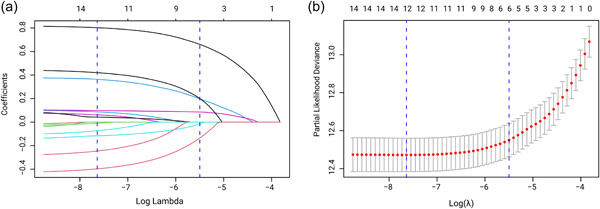
Radiotherapy‐induced cardiac mortality in patients with esophageal cancer: Least absolute shrinkage and selection operator (LASSO) regression analysis. (a) For 16 candidate variables, minimum absolute shrinkage and selection coefficients are plotted, in which six variables with non‐zero coefficients are selected as the best predictors. (b) Ten‐fold cross‐validation via minimum criteria was used in the tuning parameter (λ) selection of the LASSO model.

To identify risk factors for cardiac death after EC irradiation, a LASSO regression model was utilized. After randomizing data 7:3 into development and validation groups, most included variables were fairly evenly distributed between the two groups. Analysis of the entire data set revealed that the six risk factors most associated with prognosis were age, surgery, year of diagnosis, sequence of surgery and radiotherapy, chemotherapy, and number of tumors (Figure 3).

### 3.3 Construction and validation of nomogram models

**Figure 4:**
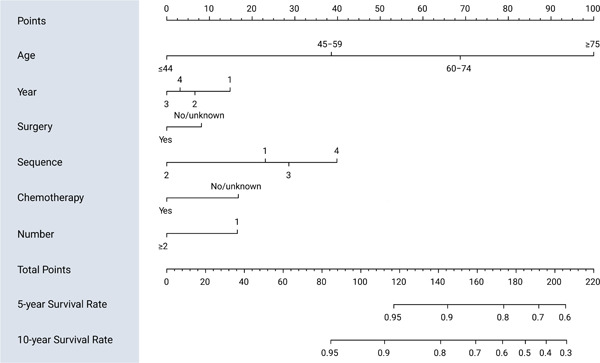
Nomograms for predicting 5‐ and 10‐year survival rates. Year: 1, 2000–2005; 2, 2006–2010; 3, 2011–2015; 4, 2016–2018. Sequence: 1, radiation before surgery; 2, intraoperative radiation; 3, radiation after surgery; 4, unknown.

The results were analyzed using LASSO regression, age (correlation coefficient *r* = 0.646), surgery (*r* = 0.184), year of diagnosis (*r* = −0.0003), sequence of surgery and radiotherapy (*r* = 0.085), chemotherapy (*r* = 0.172), and a number of tumors (*r* = −0.107) were used to construct the 5‐ and 10‐year survival likelihood nomograms for the risk of cardiac death in patients receiving radiotherapy for EC (Figure 4). The variance analysis results of each variable's influence on the model are shown in Table 2.

**Table 2:** The variance results for the effect of each risk factor on the model.

Risk factor	Chi‐Square	*df*	*p‐*Value
Age	248.26	3	<0.0001
Year of diagnosis	19.65	3	0.0002
Surgery	0.76	1	0.3820
Sequence	4.19	3	0.2415
Chemotherapy	22.86	1	<0.0001
Number	31.12	1	<0.0001
TOTAL	522.71	12	<0.0001

**Figure 5:**
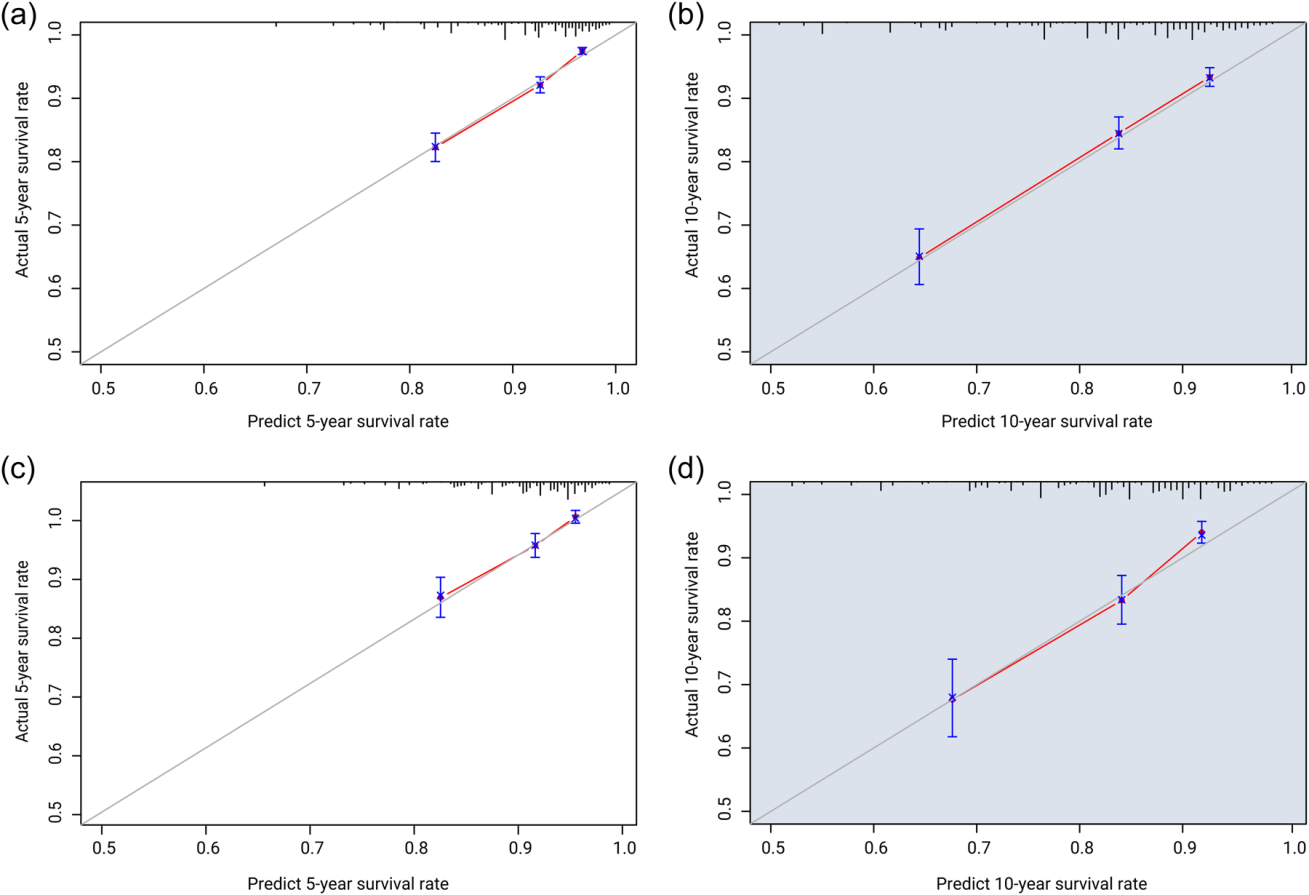
Prognostic nomogram calibration graph for each group. The concordance index (C‐index) of the development group is 0.708, and that of the validation group is 0.679, suggesting that the model has high accuracy. (a) Five‐ and (b) 10‐year calibration plots for the development group. (c) Five‐ and (d) 10‐year calibration plots for the validation group.

The C‐indexes of the nomogram models were 0.708 for the development group and 0.679 for the validation group. In both cohorts, calibration curves for 5‐ and 10‐year risk of cardiac death after radiation for EC demonstrated excellent correlation between predicted and observed values (Figure 5).

**Figure 6:**
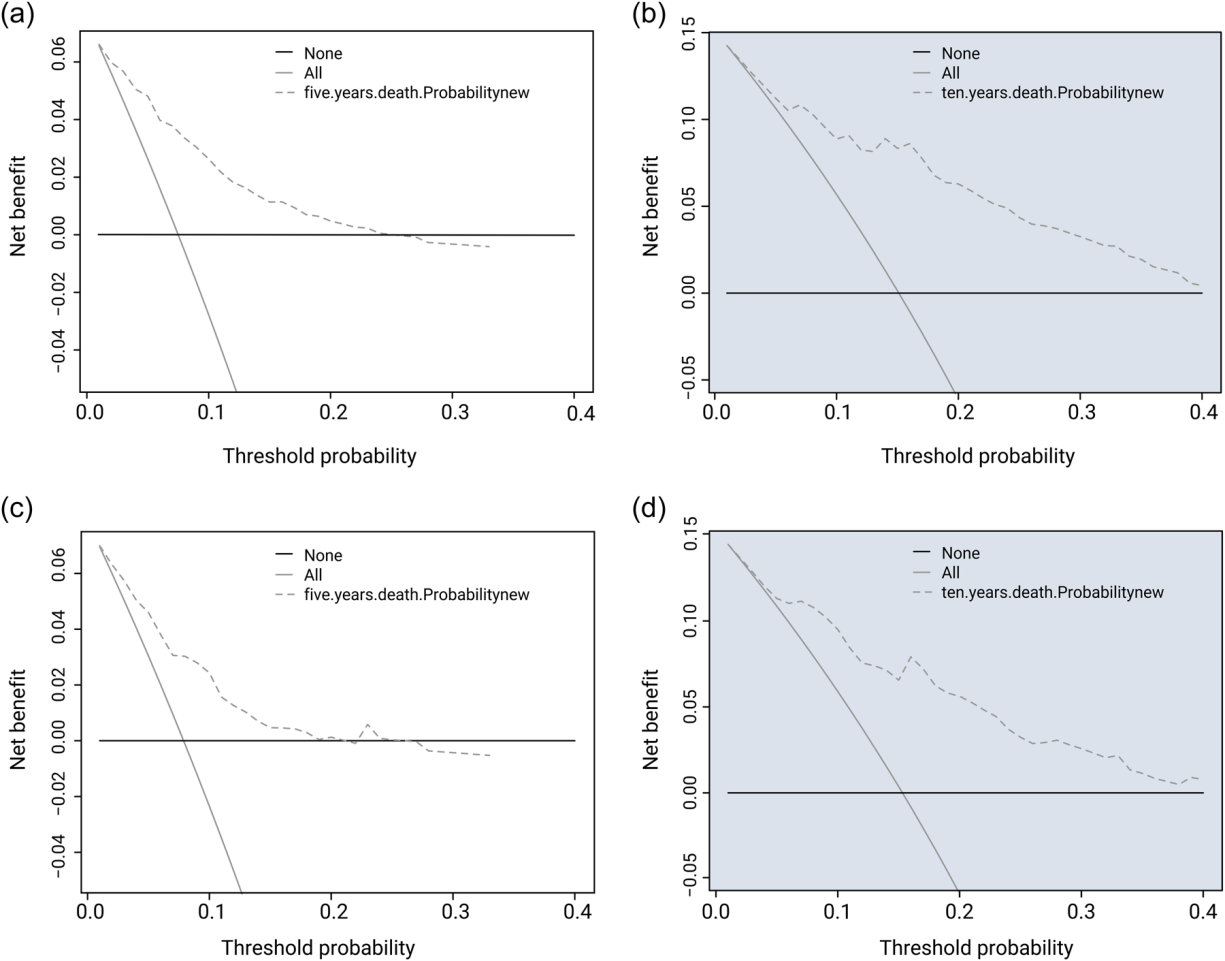
Cardiac death risk in patients receiving radiation therapy for esophageal cancer: Prognostic Decision Curve Analysis (DCA). DCA of the model's clinical value in prediction of (a) 5‐ and (b) 10‐year survival rate for the development group. DCA of the model's clinical value in prediction of (c) 5‐ and (d) 10‐year survival rate for the validation group.

**Figure 7:**
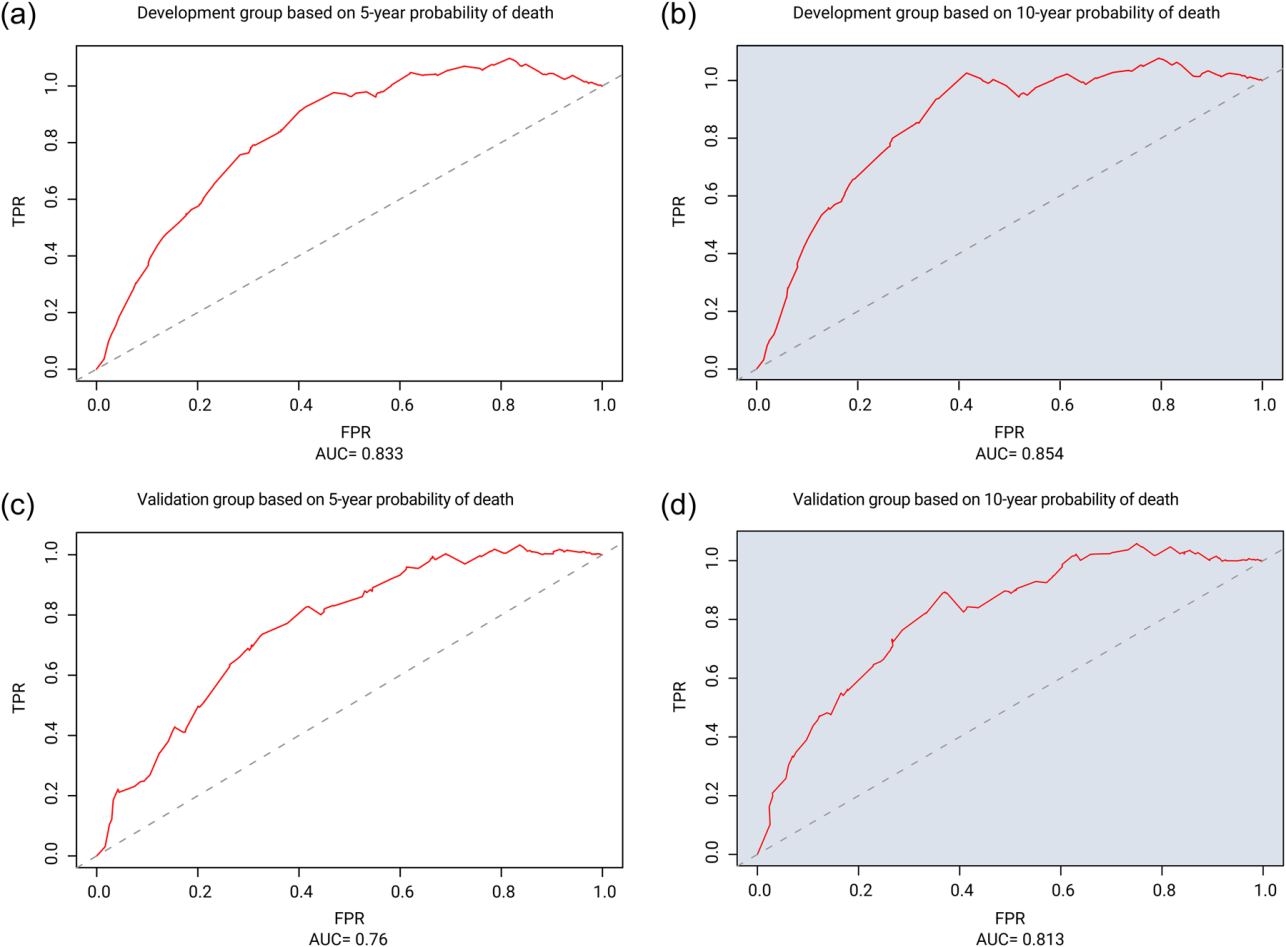
Cardiac death risk in esophageal cancer patients receiving radiation therapy depicted by receiver operating characteristic curves (ROC). (a) 5‐ and (b) 10‐year ROC curves for the development group. (c) 5‐ and (d) 10‐year ROC curves for the validation group. FPR, false positive rate; TPR, true positive rate.

Objective DCA of the model's clinical value demonstrated that our model can accurately predict the probability of cardiac death at 5 and 10 years in patients receiving EC radiation (Figure 6). We generated AUC values and performed survival analyses for the two groups using Cox regression models. The 5‐ and 10‐year AUC values of the development group (Figure 7a,b) and validation group (Figure 7c,d) were 0.833 and 0.854, and 0.76 and 0.813, respectively.

### 3.4 K‐M survival analysis based on death probability

**Figure 8:**
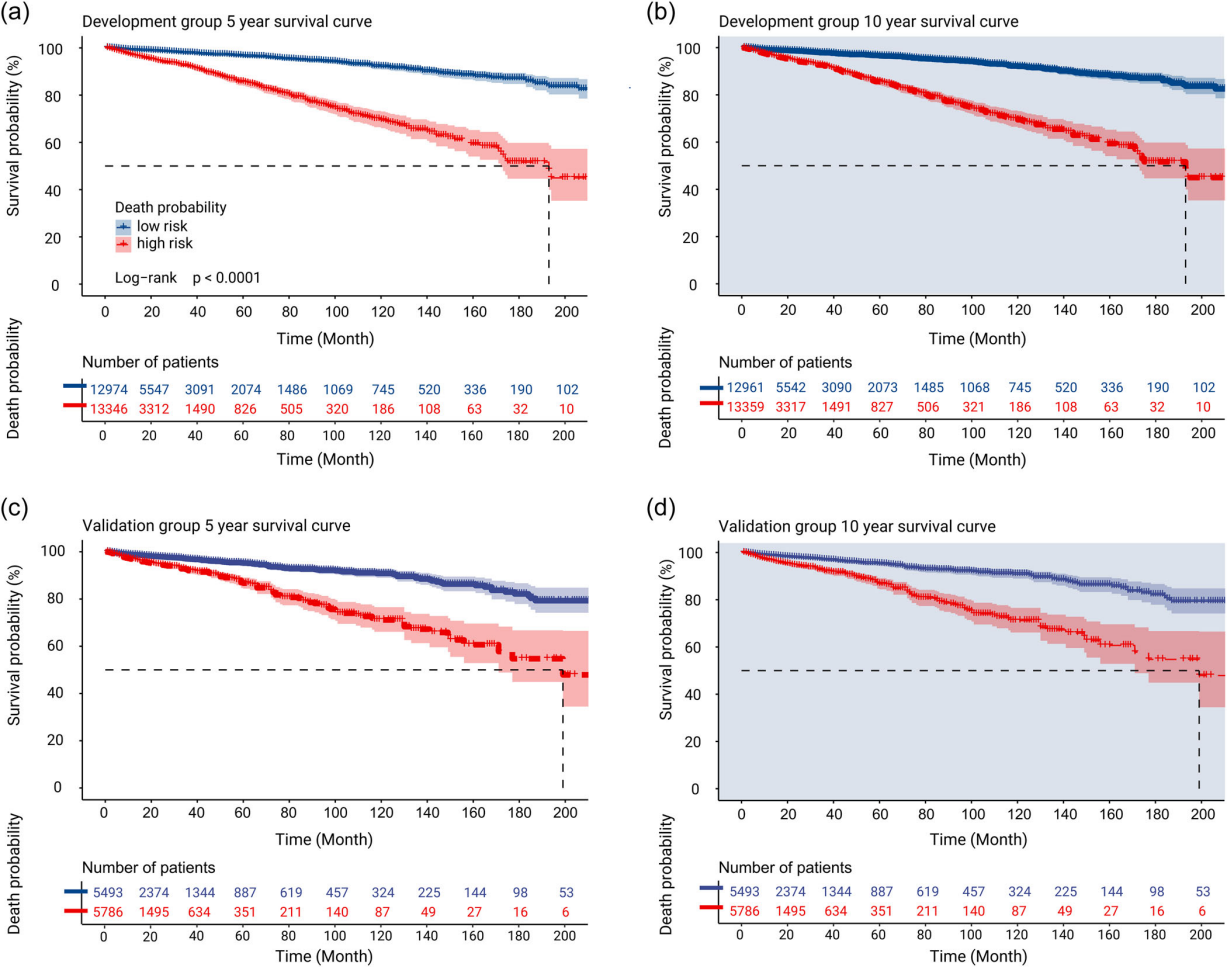
The probability of death estimated by Kaplan–Meier survival analysis. (a) 5‐ and (b) 10‐year survival curves with the cut‐off points of the development group. (c) 5‐ and (d) 10‐year survival curves with the cut‐off points of the validation group.

The optimal cut‐off points were calculated according to age, year of diagnosis, surgery, sequence of surgery and radiotherapy, chemotherapy and number of tumors, using the probability of death. Two groups were formed according to the probability of death values. The ideal probability cut‐offs for mortality at 5 and 10 years were 0.075 and 0.16 for the development group, respectively, and 0.075 and 0.1598 for the validation group, respectively. Individual survival numbers and time data were combined, and 5‐ and 10‐year K‐M survival curves were shown in Figure 8.

## 4 DISCUSSION

EC is one of the 10 most common malignancies globally [28]. Anticancer therapy can cause cardiotoxicity, and EC survivors often die of cardiovascular disease after cancer [2, 3]. Therefore, improving patient outcomes requires early detection of cardiac death risk in EC patients receiving anticancer therapy. We developed nomogram models of 5‐ and 10‐year survival probabilities to identify risk factors associated with cardiac death in EC patients treated with radiation therapy. The model was based on six predictors: age, surgery, year of diagnosis, sequence of surgery and radiotherapy, chemotherapy, and number of tumors.

In the development group, the C‐index was 0.708, and AUC values were 0.833 (95% confidence interval [CI]: 0.813–0.853, 5 years) and 0.854 (95% CI: 0.834–0.874, 10 years); the C‐index of the validation group was 0.679, and the AUC values were 0.76 (95% CI: 0.729–0.791, 5 years) and 0.813 (95% CI: 0.782–0.844, 10 years). The AUC and DCA results suggested that the model we developed has high accuracy (ROC sensitivity: 0.634, specificity: 0.806, positive likelihood ratio: 3.261, negative likelihood ratio: 0.454, positive predictive value: 0.650, negative predictive values: 0.795), and consistency in predicting the risk of cardiac death after radiotherapy for EC.

According to the current study, age is the most critical risk factor for cardiac death in individuals receiving radiation therapy for EC. The findings suggest that the risk of cardiac death rises sharply with age, especially in those over 75 years of age. These results are similar to those reported by Gharzai et al. [24], suggesting that mortality from cardiac causes after radiotherapy in patients with EC increased with age. Likewise, Frandsen et al. [23] confirmed that age was positively correlated with the occurrence of cardiac death after radiotherapy for EC, and age was linearly associated with cardiotoxicity and was a persistent risk factor for cardiotoxicity after radiotherapy in a cohort study conducted at three cancer centers in the United States. Cardiotoxicity and mortality are more prevalent in individuals over the age of 65 [19]. In EC patients treated with radiotherapy, age is an individual predictor of overall survival [29, 30].

In our study, the sequence of surgery and radiotherapy was the second most important risk factor for cardiac death in patients receiving radiotherapy for EC. We found in the literature that in patients with EC, radiation therapy given before or after surgery increases the risk of cardiac death. For example, two studies [31, 32] have found that after preoperative radiotherapy, there was a 36% increase in late cardiac events and a 14.9% increase in atrial fibrillation, and the risks of late cardiovascular events and atrial fibrillation correlated with age and radiotherapy dose. Another study [33] showed that postoperative radiotherapy also increased cardiovascular events in patients with EC. Our findings that preoperative radiotherapy was associated with a lower incidence of cardiac death compared with postoperative radiotherapy in patients with EC are consistent with a study in rectal cancer [34]. In addition, Qiu et al. [35] showed that for patients with metastatic EC, preoperative radiotherapy may have a higher survival benefit than postoperative radiotherapy. In theory, preoperative radiotherapy has the advantages of low dose, promoting tumor downstaging, improving tumor resectability and eliminating micrometastases [36]. Furthermore, surgery followed by radiotherapy had the greatest benefit in the treatment of locally advanced EC [37]. Postoperative radiotherapy may require 15–20 Gy higher doses than preoperative radiotherapy to achieve similar efficacy [38], and patients who received preoperative radiotherapy may also be considered in combination with other treatments, such as postoperative adjuvant chemotherapy.

The combination of radiotherapy and chemotherapy has improved the survival rate of patients with EC. However, it may cause cardiac death in patients with EC, particularly those with underlying cardiac problems [39]. Patients who received radiotherapy and chemotherapy were more likely to develop cardiac problems, such as cardiac arrhythmias and heart failure, than those who received radiotherapy alone [28]. Radiotherapy alters the microvascular and macrovascular environment, accelerates atherosclerosis, and leads to cardiac fibrosis [40]. The second most popular cardiotoxic drug with radiosensitizing properties is 5‐FU, which is also used as a chemotherapeutic drug in the treatment of solid tumors. Ischemia and direct damage to cardiomyocytes are the two main consequences of 5‐FU‐induced cardiotoxicity. Combined radiotherapy and 5‐FU in patients with EC may exacerbate cardiotoxicity and reduce cardiac function [41, 42, 43, 44].

Patients with recently diagnosed EC had a lower risk of dying from cardiac disease after radiotherapy than those with earlier diagnosed EC. This may be related to the use of better and newer radiation techniques and cardioprotective therapy [39]. The number of tumors and the accessibility to surgery were additional risk factors for cardiac death in patients receiving radiotherapy for EC. Those who underwent surgery without comorbidities were at greater risk of dying from heart disease than those who underwent surgery with comorbidities [23].

The nomogram developed in this study was performed using a large sample, had moderate predictive power, and provided new insights into the risk of cardiac death after radiotherapy for EC. However, this study has several limitations. First, this retrospective study was based on data from public databases, which means it has limitations and potential bias. Second, we only validated the study data internally. The results of this study require external validation in the real world. Third, the SEER database does not contain information on radiation therapy doses. Therefore, the association between dose and cardiac death could not be analyzed. Fourth, the SEER database only reports whether patients received radiotherapy and chemotherapy, but does not give specific chemotherapy regimens, and does not mention other treatments, such as immunotherapy, targeted therapy, and so on, so we cannot get more information. Fifth, no information was available on whether patients had underlying cardiac disease, received cardioprotective therapy, or had comorbidities. This may lead to an exaggerated likelihood of radiotherapy causing cardiac death in patients with EC.

According to the 2017 American Society of Clinical Oncology (ASCO) guidelines, patients receiving more than 30 Gy of thoracic radiation therapy are at increased risk of radiation‐induced cardiac injury. ASCO recommends that physicians promptly monitor for cardiotoxicity and assess baseline cardiac risk factors in this high‐risk population [45]. Serum biomarkers [46, 47, 48], echocardiography [49, 50, 51], advanced cardiovascular imaging [52, 53], and artificial intelligence‐assisted diagosis [54, 55] facilitate early identification of cardiovascular toxicity associated with cancer therapy. Efforts should be made to improve the oncologists' and patients’ awareness of cancer treatment‐related cardiotoxicity, further explore personalized treatment options, and combine the above tools for early screening and follow‐up of cardiotoxicity. Patients with EC should also begin cardioprotective therapy as soon as possible to minimize the threat of cardiotoxicity and cardiovascular death [56].

## 5 CONCLUSIONS

Radiotherapy is a necessary cancer treatment, but it increases the probability of cardiovascular toxicity and death. This should always be considered when determining the likelihood of long‐term survival in EC patients receiving radiotherapy. In the current study, a nomogram was developed and validated to accurately predict cardiac death after radiotherapy in EC patients. Risk factors such as age, year of diagnosis, surgery, sequence of surgery and radiotherapy, chemotherapy, and number of tumors were included in the nomogram. By early detection of cardiac death risk in patients receiving radiation therapy for EC, our nomogram can help improve patient outcomes and overall survival by reducing the risk of cardiac death.

## AUTHOR CONTRIBUTIONS

**Xinfang Lv**: Conceptualization (lead); writing—original draft (lead). **Xue Wu**: Conceptualization (equal). **Kai Liu**: Conceptualization (equal). **Xinke Zhao**: Conceptualization (equal). **Chenliang Pan**: Formal analysis (equal). **Jing Zhao**: Formal analysis (equal). **Juan Chang**: Data curation (equal). **Huan Guo**: Data curation (equal). **Xiang Gao**: Data curation (equal). **Xiaodong Zhi**: Data curation (equal). **Chunzhen Ren**: Data curation (equal). **Qilin Chen**: Data curation (equal). **Hugang Jiang**: Writing—review & editing (equal). **Chunling Wang**: Writing—review & editing (equal). **Ying‐Dong Li**: Conceptualization (equal).

## CONFLICT OF INTERESTS STATEMENT

The authors declare no conflict of interest.

## ETHICS STATEMENT

Through reference number 17315‐Nov2020, the National Cancer Institute of the United States granted us access to the research data in the SEER program. Approval was waived by the local ethics committee as the SEER data were deidentified and made available to this study.

## INFORMED CONSENT

Not applicable.

## Data Availability

Data for this study were downloaded from SEER plus.
